# Abstract not submitted for online publication

**DOI:** 10.1186/1742-4690-8-S2-P28

**Published:** 2011-10-03

## Abstract not submitted for online publication

Abstract not submitted for online publication

